# Risk Factors for Anemia in Patients with Chronic Renal Failure: A Systematic Review and Meta-Analysis

**DOI:** 10.4314/ejhs.v30i5.23

**Published:** 2020-09

**Authors:** Wondimeneh Shibabaw Shiferaw, Tadesse Yirga Akalu, Yared Asmare Aynalem

**Affiliations:** 1 Department of Nursing, College of Health Science, Debre Berhan University, Ethiopia; 2 Department of Nursing, College of Health Science, Debre Markos University, Ethiopia

**Keywords:** Anemia, chronic kidney injury, chronic kidney disease, chronic renal insufficiency

## Abstract

**Background:**

Anemia in patients with chronic kidney disease presents significant impacts on patients, the health-care system and financial resources. There is a significant variation in the primary studies on risk factors of anemia in this patient population across the globe. Therefore, this study aimed to identify the risk factors of anemia among chronic kidney disease patients at the global level.

**Methods:**

PubMed, Scopus, African Journals Online, Web of Science and Google Scholar were searched and complemented by manual searches. A Funnel plot and Egger's regression test were used to determine publication bias. DerSimonian and Laird random-effects modes were applied to estimate pooled effect sizes, odds ratios, and 95% confidence interval across studies. Analysis was performed using STATA™ Version 14 software.

**Result:**

A total of 28 studies with 24,008 study participants were included in this study. Female sex (AOR= 1.36; 95% CI 1.11, 1.67), stage 5 CKD (AOR = 13.66; 95% CI: 5.19, 35.92), body mass index ≥ 30 kg/m^2^ (AOR = 0.51; 95% CI: 0.29, 0.91), comorbidities (AOR = 2.90; 95% CI: 1.68, 5.0), proteinuria 3^+^(AOR = 3.57; 95% CI: 1.03, 12.93), hypocalcemia (AOR=3.61, 95%CI: 1.56–8.36), and iron therapy (AOR: 0.59; 95% CI:0.31, 0.98) were significantly associated with anemia of chronic kidney disease.

**Conclusion:**

Female sex, stage 5 CKD, body mass index ≥ 30 kg/m^2^, comorbidity, and hypocalcemia were found to be significantly associated with anemia of chronic kidney disease. Therefore, situation-based interventions and country context-specific preventive strategies should be developed to reduce the risk factors of anemia in patients with chronic renal failure.

## Introduction

Chronic Kidney Disease (CKD) is a rising global health problem, defined as kidney damage or glomerular filtration rate (GFR) of less than 60 mL/min/1.73 m^2^ for three months or more, irrespective of the cause or evidence of kidney damage (1,2). Chronic kidney disease is emerging as a complex global health problem with a huge economic burden both on the affected family of patients and on the healthcare delivery system (3).

Anemia is a serious complication of CKD and has significant adverse outcomes (4). When diseased kidney loses its ability to produce the erythropoietin essential to the production of hemoglobin, anemia is developed(5). Anemia with CKD is defined as a situation in which the concentration of hemoglobin (Hb) in the blood is below the mean Hb level (6). According to the Kidney Disease Improving Global Outcomes (KDIGO) Anemia Work Group, anaemia in CKD occurs when the Hb level is <13 g/dL for men and <12 g/dL for women (7). An estimated glomerular filtration rate (eGFR) of less than 60 ml/min/1.73m^2^ is the best indicator for the investigation of anemia in CKD patients (8).

Large differences have been reported on the magnitude of anemia in patients with CKD across studies. For instance, reports showed an anemia prevalence of 47.7% in the USA (9), 39.36% in India (10), 97.8% in Brazil (11), 51.5% in China (12), 79% in Cameroon (13), 43.18% in South Africa (14), and 64.5% in Ethiopia (15). In addition, African Americans had a 3-fold increased likelihood of anemia compared with whites (16).

Although the primary cause of anemia in patients with CKD is the inadequate production of erythropoietin by the kidneys to support erythropoiesis (17), there is also the result of a complex interplay between patient-specific attributes including diabetes with or without nephropathy (DN)(12), advanced CKD stages (12,14,18,19), nutritional deficiency (iron, folic acid, and vitamin B12) (20), diabetes mellitus (14), hematological disorders, not taking iron supplements, respiratory disorders (18), body mass index (BMI)<18.5 kg/m^2^, history of hemodialysis and rural residence (15), smoking, and reduced serum albumin (21).

The potential adverse clinical outcomes of anemia in CKD patients include: cognitive impairment, angina, cardio-renal anemia syndrome (22), left ventricular hypertrophy (LVH) (23), higher healthcare costs and reduced quality of life (24,25), increased hospital admission rate (26), worsening CKD (27), accelerated progression of heart disease (4,27), and increased mortality (27,28). However, some studies have shown that early identification and prompt treatment of anemia through near normalization of hemoglobin and iron levels in CKD patients is associated with reduced renal disease progression, as well as improved energy, work capacity, health-related quality of life, cognitive function, and cardiac function (4,29). In addition, optimizing the Hbor hematocrit value before initiating dialysis may reduce mortality (25). Likewise, studies demonstrate that positive correlations between increases in hemoglobin levels and quality of life measures were reported (24,30,31).

Different primary studies worldwide show the risk factors of anemia as a health issue on the continent. However, variation was observed among these studies. Therefore, this systematic review and meta-analysis aimed to identify risk factors for anemia in patients with CKD.

## Methods

**Literature search strategy**: Electronic databases such as PubMed, Google Scholar, African journals Online, Scopus, Web of Science, and Psyinfo were systematically searched. Grey literature such as surveillance reports, academic dissertations and conference abstracts was examined. In addition, the reference lists of the included articles were hand-searched to identify any potentially relevant articles. This search involved articles published from inception to February 13, 2020. The searches were restricted to full texts, free articles, human studies, and English language publications. Endnote X 8.1 reference manager software was used to collect and organize search outcomes and to remove duplicate articles. The search was conducted using the following terms and phrases: “anaemia”, “risk factors”, “associated factors”, “chronic kidney injury”, “chronic kidney disease”, “chronic renal insufficiency”, “global”, “international” and “list of continent”. Boolean operators such as “AND” and “OR” were used to combine search terms.

**Eligibility criteria**: Studies were included in the meta-analysis if they fulfilled the following criteria: (1). all observational studies investigating risk factors of anemia in patients with CKD, (2) articles published in peer-reviewed journals or grey literature, and (3) articles published in English from inception to 2020. Studies were excluded if: (1) they were not fully accessible, (2) they were duplicated citations, and (3) they possessed a poor quality score as per the stated criteria.

**Data extraction and quality assessment**: Two independent investigators screened the titles and abstracts of all potential studies. Data were extracted from each of these studies using the standardized data extraction format prepared in a Microsoft™Excel worksheet by the three authors independently. For each included article, we extracted data regarding the name(s) of the author(s), year of publication, study area, study design, sample size, data collection year, sampling technique, diagnostic criteria used for anemia, reported prevalence with its 95% Confidence Interval (CI) and information regarding the associated factors. The quality of each included study was assessed using the Newcastle-Ottawa scale (NOS)(32). Studies were included in the analysis if they scored ≥5 out of 10 points in three domains of ten modified NOS components for observational studies. Any disagreements at the time of data abstraction were resolved by discussion and consensus (Supplementary file 1). In addition, the risk of bias of selected articles was assessed using the risk of bias tool for prevalence studies developed by Hoy et al. Two authors carried out the risk of bias assessment of the included studies independently.

**Statistical analysis**: To obtain risk factors for anemia in patients with CKD, a meta-analysis using the random-effects DerSimonian and Laird model was performed (33). Cochran's Q chisquare statistics and I^2^ statistical tests were conducted to assess the random variations between primary studies (34).To investigate the sources of heterogeneity, meta-regression and subgroup analyses were performed. Potential publication bias was assessed by visually inspecting funnel plots and objectively using the Egger bias test (35). Sensitivity analysis was used to see the effect of a single study on the overall effect estimation. Meta-analysis was performed using STATA™ version 14 statistical softwarefor Windows™ (36).

**Presentation and reporting of results**: The results of this review were reported based on the Preferred Reporting Items for Systematic Review and Meta-Analysis statement (PRISMA) guidelines (37). The entire process of study screening, selection, and inclusion was described with the aid of a flow diagram. The results are presented using forest plots and summary tables.

## Results

**Search results**: The search strategy identified a total of 1,884 articles. Approximately 1,879 studies were found from five international databases and the remaining 5 were identified through a manual search. The databases included PubMed (948), Scopus (156), Google Scholar (572), African Journals Online (171), and Web of Science (32). After excluding duplicate publications, 949 articles remained. Approximately 789 articles were excluded after reading the titles and abstracts based on the predefined eligibility criteria. Out of them, 160 articles were included and screened for further assessment. Finally, 28 articles were included in the analysis.

**Baseline characteristics of the included studies**: In the current meta-analysis, a total of 28 studies with 24,008 study participants were included to identify risk factors of anemia among CKD patients. Regarding study design, most (75%) of the studies included were cross-sectional. The number of participants per study ranged from 39 to 5,222. Risk factors of anemia patients with CKD were obtained from various areas across the globe. Twelve studies involved participants from Africa (13–15,38–46), nine from Asia (18,47–53), four from Europe (28,54–56), and three from America (9,11,57).With respect to the tools used to assess anemia in CKD patients, eleven studies (11,12,15,38,43–45,48,49,52,57) used the WHO definition, nine studies (13,14,42,46,47,51,53,55,56) used the Kidney Disease Outcome Quality Initiative, and three studies (28,39,41) did not specify the tool they used. The quality score of each primary study, based on the Newcastle-Ottawa quality score assessment, was moderate to high for all the 28 articles assessed (Table 1).

**Table 1:** Baseline characteristics of the included studies

Author	Pub.year	Country, continent	Sample Size	The diagnosis of anemia	Risk factors	Quality score
Adera H., et al. [15]	2019	Ethiopia, Africa	251	WHO definition	rural residence BMI hemodialysis history	7
Afshar R., et al. [48]	2010	Iran, Asia	100	NKF-K/DOQI	BUN, Hb concentration, creatinine clearance	6
Akinola OI., et al. [40]	2018	Nigeria, Africa	55	WHO definition	aging, female gender, history of DM, declining eGFR	6
Akinsola A., et al. [41]	2000	Nigeria, Africa	39	Not Specified	severity of the renal failure	6
Bueno CS., et al. [11]	2013	Brazil, South America	45	WHO definition	ferritin, creatinine, urea under	6
Ijoma C., et al. [42]	2010	Nigeria, Africa	364	<12mg/dl	chronic glomerulonephritis HIV, chronic pyelonephritis	7
Delestudio., et al. [55]	2014	Spain, Europe	504	NKF	Stage of CKD	8
Di Iorio B., et al. [56]	2007	Italy, Europe	2,746	K-DOQI	Gender, years of dialysis, BMI, serum albumin, and calcium	8
Elgari MM., et al. [38]	2019	Sudan, Africa	100	Not Specified	erythropoietin and iron therapy	6
Han JS., et al. [49]	2015	Korean, Asia	1,456	WHO definition	Albuminuria	8
Haupt L., et al. [43]	2016	South Africa, Africa	49	K-DOQI	Iron therapy	6
Juma A [44]	2012	Tanzania, Africa	100	WHO definition	EPO level	7
Jungers PY., et al. [57]	2002	France, Europe	403	K-DOQI	Epoetin therapy Creatinine clearance	8
Kaze FF., et al. [13]	2015	Cameroon, Africa	95	K-DOQI	Erythropoietin treatment	6
Li Y., et al. [12]	2016	China, Asia	2,420	WHO definition	advancing CKD stage, iron therapy, chronicglomerulonephritis	8
Lau et al[18]	2015	Singapore, Asia	457	KDIGO	stage 5 CKD, hematological and respiratory disorders	7
Maïz HB., et al. [45]	2002	Tunisian, Africa	304	WHO definition	History of dialysis	7
McClellan W., et al.[9]	2004	United state, North America	5,222	<12mg/dl	EPO therapy	8
Meremo AJ., et al. [46]	2017	Tanzania, Africa	792	WHO definition	blood loss, eGFR, serum creatinine level, and Urea level	7
Nalado AM., et al. [14]	2019	South Africa, Africa	397	K-DOQI	CKD stage V, Diabetes Mellitus, ethnic disparity	6
Raji YR, et al. [47]	2020	Nigeria, Africa	314	K-DOQI	Female gender severity of CKD	6
Ryu S-R., et al. [50]	2017	Korean, Asia	2,198	WHO definition	CKD stages, body mass index (BMI), smoking, leukocyte count, serum albumin, iron markers, calcium, and phosphorus concentration	8
Salman M., et al. [51]	2016	Malaysia, Asia	615	KDIGO	advanced stages of CKD, iron therapy	8
Shaheen FA., et al. [52]	2011	Saudi, Asia	250	K-DOQI	Advanced stages of CKD, iron therapy	7
Stauffer ME., etal[39]	2014	United state, North America	1,691	NAAC and WHO	Advanced stage of CKD	8
Stirnadel-Farrant HA., et al. [28]	2018	England, Europe	266	Not Specified	advanced CKD, diabetes mellitus, peripheral vascular disease	7
Suega K., et al. [53]	2005	Indonesia, Asia	52	WHO definition	Low serum folic acid	6
Vikrant S., et al. [54]	2019	India, Asia	2,723	K-DOQI	deficiency of folic acid and Vitamin B12	8

K-DOQI: Kidney Disease Outcome Quality Initiative; NKF:National Kidney Foundation;NAAC: National Anemia Action Council; WHO: World Health Organization;KDIGO: Kidney Disease: Improving Global Outcomes

## Factors Associated with Anemia Among CKD Patients

**Figure 2:**
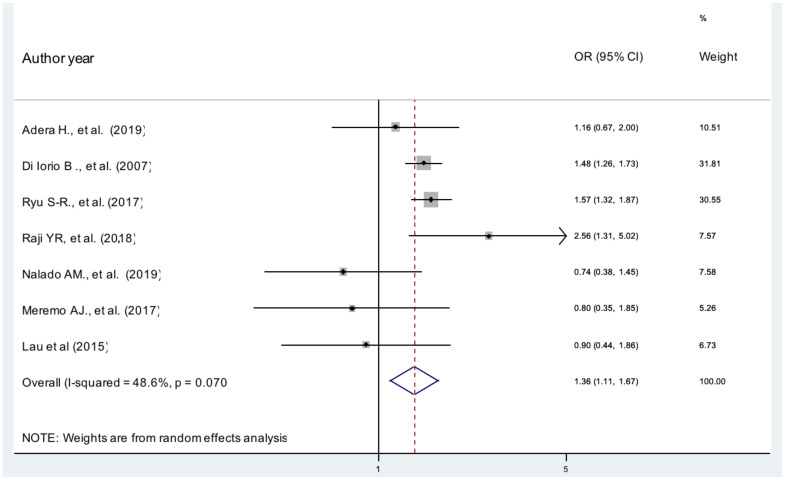
The pooled effects of sex on anemia patients with CKD

**Gender**: According to our current metaanalysis, females were 36% more likely to develop anemia in patients with CKD than male patients (AOR, 1.36, 95% CI 1.11, 1.67, I^2^ =48.6%) (Figure 2). The evidence from Egger's regression test shows that there was no publication bias (P = 0.203).

**Figure 3:**
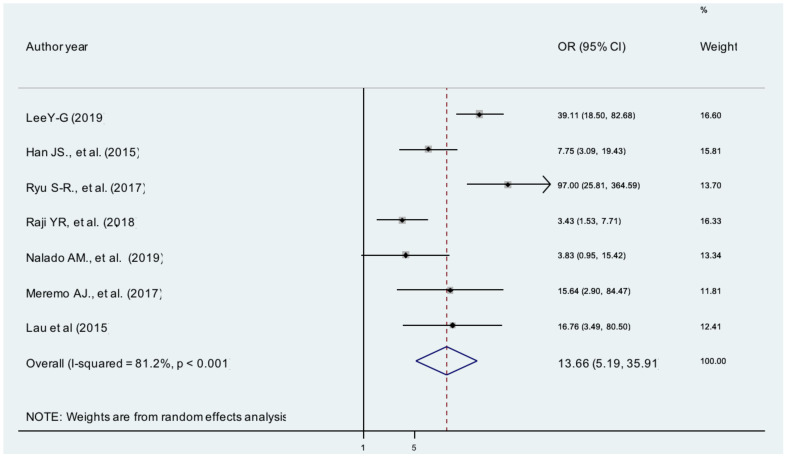
The pooled effects of stage of CKD on anemia

**Stage of CKD**: The pooled effects of seven studies showed that stage 5 CKD patients were13 times more likely to develop anemia than patients with stage 1 CKD [AOR = 13.66; 95% CI: 5.19, 35.92, I^2^ =81.2%], (Figure 3).The evidence from Egger's regression test showed that there was publication bias (P = 0.005).

**Age**: According to the currentmeta-analysis, the pooled effects of four studies (14,15,46,49) indicated that those over 50 years of age were 62% more likely to develop anemia compared to those less than 50 years old, although this association was not statistically significant (OR: 1.62 (95% CI (0.69, 3.79)). The result of the Egger's regression test showed no evidence of publication bias (P = 0.385).

**Figure 4:**
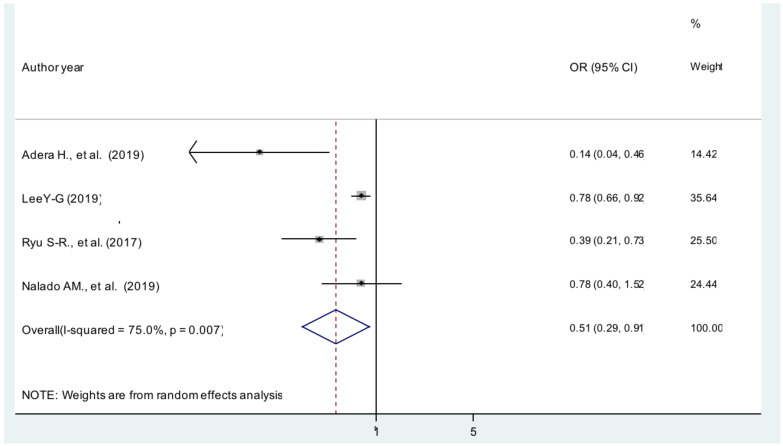
The pooled effect of body mass index on anemia patients with CKD

**Body mass index**: The current meta-analysis showed that patients with BMI ≥30 kg/m^2^ were 49% less likely to develop anemia compared with patients whose BMI was between 18.5 and 25 kg/m^2^ [AOR = 0.51; 95% CI: 0.29, 0.91, I^2^ =75%] (Figure 4). The evidence from Egger's regression test shows that there was no publication bias (P = 0.181).

**Figure 5:**
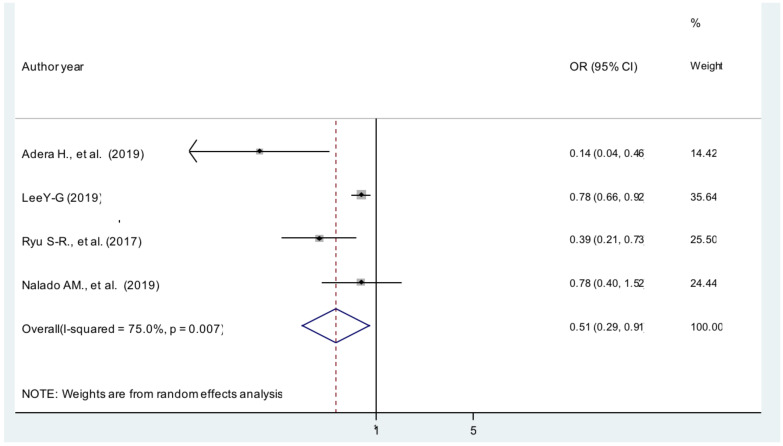
The pooled effect of comorbidities on anemia patients with CKD

**Comorbidities**: The present meta-analysis revealed that patients with comorbidities were nearly 3 times more likely to develop anemia than those with no evidence of comorbidities [AOR = 2.90; 95% CI: 1.68, 5.0, I^2^ = 86%] (Figure 5). The evidence from Egger's test shows no significant proof of publication bias (P = 0.690).

**Proteinuria**: The pooled effects of two studies (15,49) showed that those patients who had 3^+^ urine protein were 3.57 times more likely to develop anemia than patients who did not have proteinuria [AOR = 3.57; 95% CI: 1.03, 12.93, I^2^ =81.7%].

**Hypocalcaemia**: Patients with hypocalcaemia had four fold higher odds for anemia (AOR=3.61, 95%CI: 1.56–8.36, I^2^=88.3) compared with patients with normal serum calcium levels. The evidence from Egger's test shows no significant proof of publication bias (P = 0.482).

**Iron therapy**: According to the current metaanalysis, the pooled effects of two studies (18,46) indicated that those receiving iron therapy were 41% less likely to develop anemia compared to those who had not taken iron therapy (AOR: 0.59, 95% CI 0.31, 0.99). The heterogeneity test (I^2^= 20.8%) showed no significant evidence of variation across studies.

## Discussion

This study aimed to synthesize evidence on the risk factors of anemia in patients with CKD at a global level. Based on the pooled analysis of adjusted odd ratio of studies, being female, stage 5 CKD, BMI≥30 kg/m^2^, comorbidity, proteinuria 3+, hypocalcaemia, and receiving iron therapy were associated with anemia of CKD.

The current study revealed that female CKD patients were 36% more likely to develop anemia. This finding is supported by previous studies conducted in Korea (58), Australia(59), London (60), and New York (16).This would suggest that female patients had lower Hb concentrations than male patients, which likely explains why females had greater risk of developing anemia (9). Similarly, we found that those who had BMI ≥30 kg/m^2^ were 49% less likely to develop anemia of CKD compared to BMI<18.5 kg/m2. This finding was supported by other studies conducted in Korea (49); however, in contrast to a study from Australia (59).This variation might explain underweight may represent a malnourished state, which is closely related with chronic inflammation in CKD (49).

The present study showed that CKD patients with pre-existing illnesses were nearly three times more likely to develop anemia, which mirrors results from studies in Australia (59), London (60) and Malaysia (61).This finding suggests that any CKD patient who presents with comorbidities should be more closely monitored for anemia. Approprite management of comorbid illnesses may therefore reduce the odds of developing anemia.

Those patients with advanced stage of CKD (stage 5) had a significant association with a anemia, which has been previously reported in studies conducted inAustralia (59), Korea (60), New York (16) and Florida (62).This association is likely explained by the deterioration of renal function being accompanied by a reduction in erythropoietin production by the kidneys, and the loss of erythropoietin results in decreased red blood cell production that increases the risk of anemia development (5,22,63).

In the present review, proteinuria 3+ increased the risk of anemia by 57% compared with patients who do not have proteinuria. This finding is in agreement with a study conducted in Korea (58). Evidence further supports that low serum albumin due to protein malnutrition and/or inflammation is responsible for inadequate response to EPO treatment (64). In addition, it is necessary to investigate the interplay between proteinuria and the development of anemia in CKD.

Those Patients with hypocalcaemia had a significant association with anemia of CKD. This result is related to the potential for high serum calcium to favor the control of anemia via inhibition of parathyroid hormone secretion, a factor which is considered responsible for EPO resistance in hemodialysis patients (65).

We found that patients who have taken iron therapy were 41% less likely to develop anemia of CKD, which was supported by studies from Cleveland, USA (22) and China (12). In addition, a randomized control study showed beneficial erythropoietic effect of iron treatment in CKD 3–5 patients having ferritin even more than 100 ng/mL (66).

This study has clinical implications in that the high magnitude of anemia in patients with CKD should guide healthcare professionals to minimize the risk of anemia by providing guidance to the patient who could be detected in health checkups, give information about possible risk factors during routine patient care, and provide knowledge about potential risk of anemia. In addition, identifying associated risk factors may help healthcare professionals treat anemia patients with CKD during their clinical care.

There are certain limitations to this review which must be acknowledged and may inform future research. First, we only used English language published articles Second, we did not pool all predictors of anemia in patients with CKD.

In general, being female, stage 5 CKD, body mass index ≥ 30 kg/m2, comorbidity and hypocalcaemia were found to be significantly associated with anemia of chronic kidney disease. Therefore, situation-based interventions and country context-specific preventive strategies should be developed to reduce the risk factors of anemia in this patient group. In addition, this meta-analysis may help policymakers and program managers design evidence-based interventions on preventing the occurrence of anemia with CKD patient populations.

## Figures and Tables

**Figure 1:**
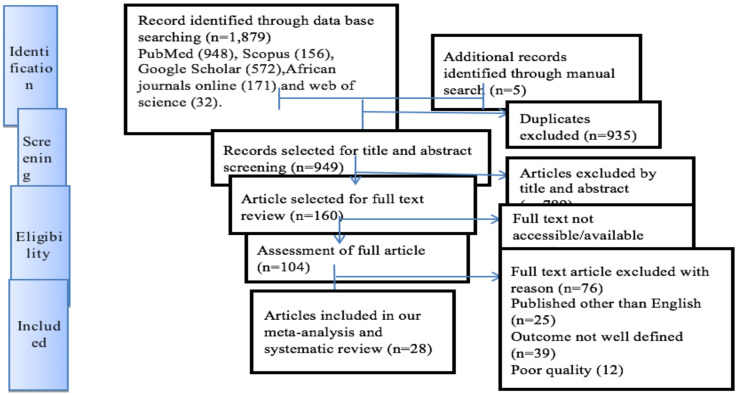
PRISMA flow chart of the study selection

## References

[1] Locatelli F, Nissenson AR, Barrett BJ, Walker RG, Wheeler DC, Eckardt KU (2008). Clinical practice guidelines for anemia in chronic kidney disease: problems and solutions. A position statement from Kidney Disease: Improving Global Outcomes (KDIGO). Kidney international.

[2] Levey AS, Eckardt K-U, Tsukamoto Y, Levin A, Coresh J, Rossert J (2005). Definition and classification of chronic kidney disease: a position statement from Kidney Disease: Improving Global Outcomes (KDIGO). Kidney international.

[3] George C, Mogueo A, Okpechi I, Echouffo-Tcheugui JB, Kengne AP (2017). Chronic kidney disease in low-income to middle-income countries: the case for increased screening. BMJ global health.

[4] Gafter-Gvili A, Schechter A, Rozen-Zvi B (2019). Iron deficiency anemia in chronic kidney disease. Acta haematologica.

[5] Robinson BE (2006). Epidemiology of chronic kidney disease and anemia. Journal of the American Medical Directors Association.

[6] Cases A, Egocheaga MI, Tranche S, Pallarés V, Ojeda R, Górriz JL (2018). Anemia of chronic kidney disease: Protocol of study, management and referral to Nephrology. Nefrología (English Edition).

[7] McMurray JJ, Parfrey PS, Adamson JW, Aljama P, Berns JS, Bohlius J (2012). Kidney disease: Improving global outcomes (KDIGO) anemia work group. KDIGO clinical practice guideline for anemia in chronic kidney disease. Kidney International Supplements.

[8] Padhi S, Glen J, Pordes BA, Thomas ME (2015). Management of anaemia in chronic kidney disease: summary of updated NICE guidance. BMJ.

[9] McClellan W, Aronoff SL, Bolton WK, Hood S, Lorber DL, Tang KL (2004). The prevalence of anemia in patients with chronic kidney disease. Current medical research and opinion.

[10] Muniyandi D, Shanmugam N, Ramanathan K, Vijayaraghavan B, Padmanabhan G (2016). Prevalence of Iron Deficiency Anemia among Chronic Kidney Disease Patients in Kaveri Delta Region, Tamilnadu, India. Journal of Advances in Medicine and Medical Research.

[11] Bueno CS, Frizzo MN (2014). Anemia in chronic kidney disease in a Hospital in the Northwest region to the State of Rio Grande do Sul. Brazilian Journal of Nephrology.

[12] Li Y, Shi H, Wang W-M, Peng A, Jiang GR, Zhang J-Y (2016). Prevalence, awareness, and treatment of anemia in Chinese patients with nondialysis chronic kidney disease: First multicenter, cross-sectional study. Medicine.

[13] Kaze FF, Kengne A, Mambap AT, Halle M-P, Mbanya D, Ashuntantang G (2015). Anemia in patients on chronic hemodialysis in Cameroon: prevalence, characteristics and management in low resources setting. African health sciences.

[14] Nalado AM, Mahlangu JN, Waziri B, Duarte R, Paget G, Olorunfemi G (2019). Ethnic prevalence of anemia and predictors of anemia among chronic kidney disease patients at a tertiary hospital in Johannesburg, South Africa. International journal of nephrology and renovascular disease.

[15] Adera H, Hailu W, Adane A, Tadesse A (2019). Prevalence Of Anemia And Its Associated Factors Among Chronic Kidney Disease Patients At University Of Gondar Hospital, Northwest Ethiopia: A Hospital-Based Cross Sectional Study. International journal of nephrology and renovascular disease.

[16] McFarlane SI, Chen S-C, Whaley-Connell AT, Sowers JR, Vassalotti JA, Salifu MO (2008). Prevalence and associations of anemia of CKD: Kidney early evaluation program (KEEP) and national health and nutrition examination survey (NHANES) 1999–2004. American Journal of Kidney Diseases.

[17] Ramanath V, Gupta D, Jain J, Chaudhary K, Nistala R (2012). Anemia and chronic kidney disease: making sense of the recent trials. Reviews on recent clinical trials.

[18] Lau BCV, Ong KY, Yap CW, Vathsala A, How P (2015). Predictors of anemia in a multiethnic chronic kidney disease population: a case-control study. Springerplus.

[19] Azab AE, Elsayed ASI (2017). Correlation between chronic kidney diseases and hematolgical data in Sabratha hospital in Libya. Asian J Pharm Clin Res.

[20] Abensur H, Bastos M, Canziani M (2006). Current aspects of Anemia in chronic kidney disease. J Bras Nefrol.

[21] Kang E, Han M, Kim H, Park SK, Lee J, Hyun YY (2017). Baseline general characteristics of the Korean chronic kidney disease: report from the KoreaN Cohort Study for Outcomes in Patients With Chronic Kidney Disease (KNOWCKD). Journal of Korean medical science.

[22] Taliercio JJ (2010). Anemia and chronic kidney disease: What's the connection?. Journal of Family Practice.

[23] Weiner DE, Tighiouart H, Vlagopoulos PT, Griffith JL, Salem DN, Levey AS (2005). Effects of anemia and left ventricular hypertrophy on cardiovascular disease in patients with chronic kidney disease. Journal of the American Society of Nephrology.

[24] van Nooten FE, Green J, Brown R, Finkelstein FO, Wish J (2010). Burden of illness for patients with non-dialysis chronic kidney disease and anemia in the United States: review of the literature. Journal of medical economics.

[25] Dowling TC (2007). Prevalence, etiology, and consequences of anemia and clinical and economic benefits of anemia correction in patients with chronic kidney disease: an overview. American journal of healthsystem pharmacy.

[26] Garlo K, Williams D, Lucas L, Wong R, Botler J, Abramson S (2015). Severity of anemia predicts hospital length of stay but not readmission in patients with chronic kidney disease: a retrospective cohort study. Medicine.

[27] Thorp ML, Johnson ES, Yang X, Petrik AF, Platt R, Smith DH (2009). Effect of anaemia on mortality, cardiovascular hospitalizations and end-stage renal disease among patients with chronic kidney disease. Nephrology.

[28] Stirnadel-Farrant HA, Luo J, Kler L, Cizman B, Jones D, Brunelli SM (2018). Anemia and mortality in patients with nondialysis-dependent chronic kidney disease. BMC nephrology.

[29] Hayashi T, Suzuki A, Shoji T, Togawa M, Okada N, Tsubakihara Y (2000). Cardiovascular effect of normalizing the hematocrit level during erythropoietin therapy in predialysis patients with chronic renal failure. American Journal of Kidney Diseases.

[30] Lefebvre P, Vekeman F, Sarokhan B, Enny C, Provenzano R, Cremieux P-Y (2006). Relationship between hemoglobin level and quality of life in anemic patients with chronic kidney disease receiving epoetin alfa. Current medical research and opinion.

[31] Spinowitz B, Pecoits-Filho R, Winkelmayer WC, Pergola PE, Rochette S, Thompson-Leduc P (2019). Economic and quality of life burden of anemia on patients with CKD on dialysis: a systematic review. Journal of medical economics.

[32] Shea BJ, Reeves BC, Wells G, Thuku M, Hamel C, Moran J (2017). AMSTAR 2: a critical appraisal tool for systematic reviews that include randomised or nonrandomised studies of healthcare interventions, or both. bmj.

[33] DerSimonian R, Laird N (1986). Meta-analysis in clinical trials. Controlled clinical trials.

[34] Huedo-Medina TB, Sánchez-Meca J, Marín-Martínez F, Botella J (2006). Assessing heterogeneity in meta-analysis: Q statistic or I2 index?. Psychological methods.

[35] Egger M, Davey-Smith G, Altman D (2008). Systematic reviews in health care: metaanalysis in context.

[36] StataCorp L (2015). Stata statistical software (version release 14).

[37] Moher D, Liberati A, Tetzlaff J, Altman DG (2009). Preferred reporting items for systematic reviews and meta-analyses: the PRISMA statement. Annals of internal medicine.

[38] Akinola OI, Olawumi HO, Agaba EI (2018). Anaemia and its predisposing factors in pre-dialysis chronic kidney disease patients in Jos, Nigeria. Jos Journal of Medicine.

[39] Akinsola A, Durosinmi M, Akinola N (2000). The haematological profile of Nigerians with chronic renal failure. African journal of medicine and medical sciences.

[40] Ijoma C, Ulasi I, Ijoma U, Ifebunandu N (2010). High prevalence of anemia in predialysis patients in Enugu, Nigeria. Nephrology Research & Reviews.

[41] Elgari MM, Khabour OF, Elhag H, Elmahmoud HA, Muddathir ARM (2019). Hematological indices of end-stage chronic renal failure patients in Sudan: With or without iron supplements. Pakistan journal of pharmaceutical sciences.

[42] Haupt L, Weyers R (2016). Determination of functional iron deficiency status in haemodialysis patients in central South Africa. International journal of laboratory hematology.

[43] Juma A (2012). Prevalence of anemia and its associated factors in patients with chronic kidney disease at Muhimbili national hospital.

[44] Maïz HB, Abderrahim E, Zouaghi K (2002). Anemia and end-stage renal disease in the developing world. Artificial organs.

[45] Meremo AJ, Mwashambwa MY, Masalu MB, Kapinga J, Tagalile R, Ngilangwa DP (2017). Prevalence and predictors of anaemia among patients presenting with kidney diseases at the University of Dodoma Hospital in central Tanzania. Tanzania Journal of Health Research.

[46] Raji YR, Ajayi SO, Akingbola TS, Adebiyi OA, Adedapo KS, Salako BL (2018). Assessment of iron deficiency anaemia and its risk factors among adults with chronic kidney disease in a tertiary hospital in Nigeria. Nigerian Postgraduate Medical Journal.

[47] Afshar R, Sanavi S, Salimi J, Ahmadzadeh M (2010). Hematological profile of chronic kidney disease (CKD) patients in Iran, in predialysis stages and after initiation of hemodialysis. Saudi Journal of Kidney Diseases and Transplantation.

[48] Han JS, Lee MJ, Park KS, Han SH, Yoo TH, Oh K-H (2015). Albuminuria as a risk factor for anemia in chronic kidney disease: result from the KoreaN Cohort Study for Outcomes in Patients with Chronic Kidney Disease (KNOW-CKD). PloS one.

[49] Ryu S-R, Park SK, Jung JY, Kim YH, Oh YK, Yoo TH (2017). The prevalence and management of anemia in chronic kidney disease patients: result from the KoreaN Cohort Study for Outcomes in Patients With Chronic Kidney Disease (KNOWCKD). Journal of Korean medical science.

[50] Salman M, Khan AH, Adnan AS, Sulaiman SAS, Hussain K, Shehzadi N (2016). Prevalence and management of anemia in pre-dialysis Malaysian patients: A hospital-based study. Revista da Associação Médica Brasileira.

[51] Shaheen FA, Souqiyyeh MZ, Al-Attar BA, Karkar A, Al Jazairi AMH, Badawi LS (2011). Prevalence of anemia in predialysis chronic kidney disease patients. Saudi Journal of Kidney Diseases and Transplantation.

[52] Suega K, Bakta M, Dharmayudha TG, Lukman JS, Suwitra K (2005). Profile of anemia in chronic renal failure patients: comparison between predialyzed and dialyzed patients at the Division of Nephrology, Department of Internal Medicine, Sanglah Hospital, Denpasar, Bali, Indonesia. inflammation.

[53] Vikrant S (2019). Etiological spectrum of anemia in non-dialysis-dependent chronic kidney disease: A single-center study from India. Saudi Journal of Kidney Diseases and Transplantation.

[54] del estudio MICENAS I, Cases-Amenós A, Martínez-Castelao A, Fort-Ros J, Bonal-Bastons J, Ruiz MP (2014). Prevalence of anaemia and its clinical management in patients with stages 3–5 chronic kidney disease not on dialysis in Catalonia: MICENAS I study. Nefrología (English Edition).

[55] Di Iorio B, Cirillo M, Bellizzi V, Stellato D, De Santo N, Group CDRR (2007). Prevalence and correlates of anemia and uncontrolled anemia in chronic hemodialysis patients-the Campania Dialysis Registry. The International journal of artificial organs.

[56] Jungers PY, Robino C, Choukroun G, Nguyen-Khoa T, Massy ZA, Jungers P (2002). Incidence of anaemia, and use of epoetin therapy in pre-dialysis patients: a prospective study in 403 patients. Nephrology Dialysis Transplantation.

[57] Stauffer ME, Fan T (2014). Prevalence of anemia in chronic kidney disease in the United States. PloS one.

[58] Yi S-W, Moon SJ, Yi J-J (2019). Low-normal hemoglobin levels and anemia are associated with increased risk of end-stage renal disease in general populations: A prospective cohort study. PloS one.

[59] Ng Y-H, Myers O, Shore X, Pankratz VS, Norris KC, Vassalotti JA (2019). The Association of Altitude and the Prevalence of Anemia Among People With CKD. American Journal of Kidney Diseases.

[60] Al-Khoury S, Afzali B, Shah N, Covic A, Thomas S, Goldsmith D (2006). Anaemia in diabetic patients with chronic kidney disease—prevalence and predictors. Diabetologia.

[61] Idris I, Tohid H, Muhammad NA, Rashid MRA, Ahad AM, Ali N (2018). Anaemia among primary care patients with type 2 diabetes mellitus (T2DM) and chronic kidney disease (CKD): a multicentred cross-sectional study. BMJ open.

[62] Robinson B, Artz AS, Culleton B, Critchlow C, Sciarra A, Audhya P (2007). Prevalence of anemia in the nursing home: contribution of chronic kidney disease. Journal of the American Geriatrics Society.

[63] Moore E, Bellomo R (2011). Erythropoietin (EPO) in acute kidney injury. Annals of intensive care.

[64] Madore F, Lowrie EG, Brugnara C, Lew NL, Lazarus JM, Bridges K (1997). Anemia in hemodialysis patients: variables affecting this outcome predictor. Journal of the American Society of Nephrology.

[65] Rao DS, Shih M-s, Mohini R (1993). Effect of serum parathyroid hormone and bone marrow fibrosis on the response to erythropoietin in uremia. New England Journal of Medicine.

[66] Qunibi WY, Martinez C, Smith M, Benjamin J, Mangione A, Roger SD (2011). A randomized controlled trial comparing intravenous ferric carboxymaltose with oral iron for treatment of iron deficiency anaemia of non-dialysis-dependent chronic kidney disease patients. Nephrology Dialysis Transplantation.

